# SiGe nanocrystals in SiO_2_ with high photosensitivity from visible to short-wave infrared

**DOI:** 10.1038/s41598-020-60000-x

**Published:** 2020-02-24

**Authors:** Ionel Stavarache, Constantin Logofatu, Muhammad Taha Sultan, Andrei Manolescu, Halldor Gudfinnur Svavarsson, Valentin Serban Teodorescu, Magdalena Lidia Ciurea

**Affiliations:** 1 National Institute of Materials Physics, Atomistilor 405A, 077125 Magurele, Romania; 2 School of Science and Engineering, Reykjavik University, IS-101 Reykjavik, Iceland; 3 Academy of Romanian Scientists, 050094 Bucharest, Romania

**Keywords:** Materials science, Nanoscience and technology, Optics and photonics

## Abstract

Films of SiGe nanocrystals (NCs) in oxide have the advantage of tuning the energy band gap by adjusting SiGe NCs composition and size. In this study, SiGe-SiO_2_ amorphous films were deposited by magnetron sputtering on Si substrate followed by rapid thermal annealing at 700, 800 and 1000 °C. We investigated films with Si:Ge:SiO_2_ compositions of 25:25:50 vol.% and 5:45:50 vol.%. TEM investigations reveal the major changes in films morphology (SiGe NCs with different sizes and densities) produced by Si:Ge ratio and annealing temperature. XPS also show that the film depth profile of SiGe content is dependent on the annealing temperature. These changes strongly influence electrical and photoconduction properties. Depending on annealing temperature and Si:Ge ratio, photocurrents can be 10^3^ times higher than dark currents. The photocurrent cutoff wavelength obtained on samples with 25:25 vol% SiGe ratio decreases with annealing temperature increase from 1260 nm in SWIR for 700 °C annealed films to 1210 nm for those at 1000 °C. By increasing Ge content in SiGe (5:45 vol%) the cutoff wavelength significantly shifts to 1345 nm (800 °C annealing). By performing measurements at 100 K, the cutoff wavelength extends in SWIR to 1630 nm having high photoresponsivity of 9.35 AW^−1^.

## Introduction

The increasing research efforts to incorporate different semiconductor nanocrystals (NCs), in particular NCs containing group IV elements such as Si, Ge or SiGe NCs dielectric films continued along the last decades because of the possibility to tune electrical and optical properties by varying the NCs size, composition and density^1–6^. It was shown that the matrix material has influence on the Ge NCs size, density and shape, and passivation quality of Ge NC/matrix interface^4,7^. Silicon has an absorption cutoff wavelength of about 1.1 *μ*m corresponding to the indirect band gap of 1.12 eV which makes Si photodetectors not suitable for short-wave infrared (SWIR) applications^8^. Compared with Si, Ge has a significant bigger optical absorption band edge at about 1.7 *μ*m and improved transport properties given by the smaller indirect band gap of 0.66 eV, being a good candidate for various applications with SWIR detection requirements^9,10^. For further downscaling of the devices, the continuous development of optoelectronics devices requires the implementation of new materials with improved photoelectric and optical properties. It would be advantageous for these materials to be compatible with Si technology. The devices based on SiGe represent one of the most promising candidates and would be a breakthrough that will open possibilities for the new system-on-a-chip to incorporate optoelectronic devices into Si electronics. This can be implemented if we consider SiGe alloy advantages like tunable bandgap^11,12^ and tunable lattice constant^2^ by controlling the Ge fraction in the alloy leading to lower annealing temperature, relative low cost, flexibility of technological processing, and overcoming different electronic compatibility reasons^13^. The scientific effort is spent to understand the conditions of SiGe NCs formation into dielectric matrices (e.g. SiO_2_, HfO_2_, Si_3_N_4_, Al_2_O_3_ or TiO_2_) which goes through the usual sequence of problems regarding reduction of the oxides, diffusion of Si and Ge in the oxide matrix, nucleation and growth, frequently followed by coarsening of nanocrystals due to Ostwald ripening^4,14^. In these films, the optical and electrical properties can be additionally managed by controlling the NC density and sizes that influences the carrier quantum confinement and in turn the materials properties^15–17^. Introducing SiGe NCs into oxides is beneficial for tuning the crystallization temperature, controlling the NCs size (in the case of multilayers) and minimizing the effects given by defects like recombination centers or fast leakage paths in SiGe-based films^4^. All these properties are also dependent on the matrix material. SiO_2_ is the most suitable matrix because of its compatibility with the CMOS technology and because it forms the best interface with Si substrate. These efforts are made with the purpose of improving the optoelectronic devices on Si and enabling the SiGe NCs integration in a large area of applications such as non-volatile memories^18–20^, GeSi based high-mobility transistors^21^, photoMOSFETs^22^, solar cells^23–25^, thermoelectric applications^26^ and high-performance photodetectors^7,27–30^.

For obtaining SiGe films or multilayers with targeted electric and photoelectric properties, different deposition methods as magnetron sputtering^2^, plasma enhanced chemical vapor deposition^13^, implantation^31^ or evaporation^32^ are used. The process of NCs formation in an oxide matrix is related with the deposition conditions if the deposition takes place on heated substrate^33–35^, but the films nanocrystallization can be accomplished by thermal annealing at suitable temperatures. For obtaining SiGe NCs in thin films or multilayers, annealing in 600–1055 °C range is performed^2,33,36,37^. A challenging problem to form SiGe NCS in SiO_2_ matrix is the oxygen excess that may occur during deposition process or heating treatment. This oxygen excess can create various Ge suboxides with the increase of treatment temperature, some of them being unstable as GeO gas^38,39^.

Herein, we report a detailed investigation of the films formed of SiGe NCs embedded in SiO_2_ matrix with high photosensitivity from visible (VIS) to SWIR. Firstly, the films with two different SiGe compositions(Si:Ge:SiO_2_ of 25:25:50 and 5:45:50) were deposited by magnetron sputtering and then they were nanostructured by annealing at 700, 800 and 1000 °C for SiGe NCs formation in SiO_2_ matrix. The influence of Si:Ge composition and annealing temperature on the film morphology and structure is discussed. Also, the electrical and photoconductive properties at nanoscale are analyzed in relation with morphology and structure in order to find out how they can be tuned. Thus, we obtained films with good photoresponse in VIS-SWIR, the main contribution in SWIR being given by the SiGe NCs.

## Results and Discussions

In the following we present the structure and morphology results obtained on samples S1 (25%:25%:50%) and S2 (5%:45%:50%).

**Figure 1:**
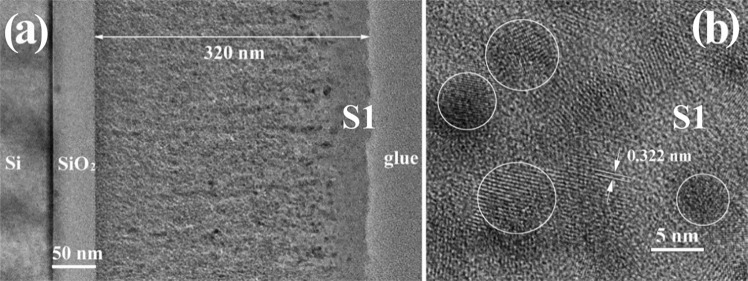
Morphology of S1 film annealed by RTA at 700 °C for 15 min: (**a**) low magnification XTEM image; (**b**) high resolution image of SiGe nanospheres.

Figure 1a shows the TEM image of the S1 film cross section after rapid thermal annealing (RTA) at 700 °C for 15 min. The S1 film has 320 nm thickness and SiO_2_ buffer layer has 50 nm. Small SiGe NCs are present in the film section, but the 40 nm top part of the film is free of NCs. The SiGe NCs have sizes between 5 and 8 nm (see Fig. 1b), and a quite uniform distribution in the SiO_2_ amorphous matrix.

**Figure 2:**
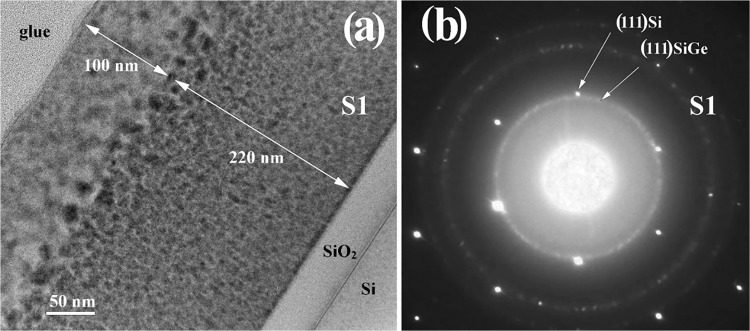
Morphology of S1 film with RTA at 800 °C, 15 min: (**a**) low magnification XTEM image, (**b**) SAED pattern of the film and Si substrate, showing the (111) Si spots and (111) ring spots of the SiGe NCs. Also, one can observe a strong amorphous component coming from the amorphous SiO_2_ matrix and the SiO_2_ buffer layer.

Figure 2a shows the low magnification XTEM image and Fig. 2b selected area electron diffraction (SAED) pattern of S1 film annealed at 800 °C for 15 min. The morphology is similar to that of 700 °C annealed film, but the top part zone without SiGe NCs is expanded to 100 nm (Fig. 2a). At the same time, at the interface between this zone (free of NCs) and the rest of the film, the SiGe NCs are bigger (from 10 to 15 nm) compared to 5–8 nm NCs from the rest of the film. The size of the SiGe NCs are similar like in the film annealed at 700 °C but the crystallization of SiGe is better as SAED pattern reveals (Fig. 2b).

**Figure 3:**
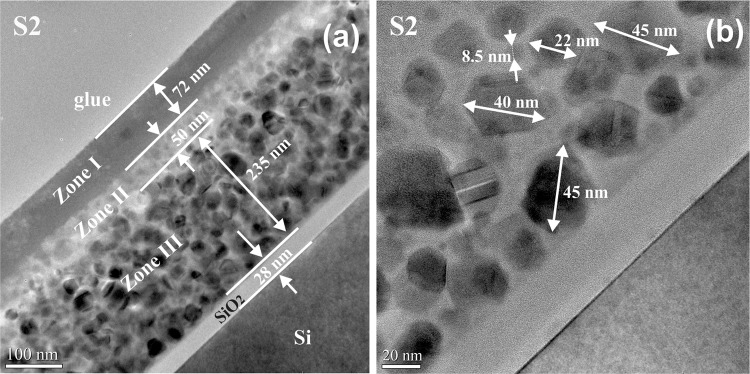
Morphology of S2 film 800 °C RTA for 10 min: (**a**) low magnification XTEM image showing three zones with different morphology; (**b**) HRTEM image of zone III at the interface with the substrate, showing the SiGe NCs embedded in the SiO_2_ matrix. The big SiGe NCs (over 30 nm) are facetted and contain nanotwins and stacking faults defects.

**Figure 4:**
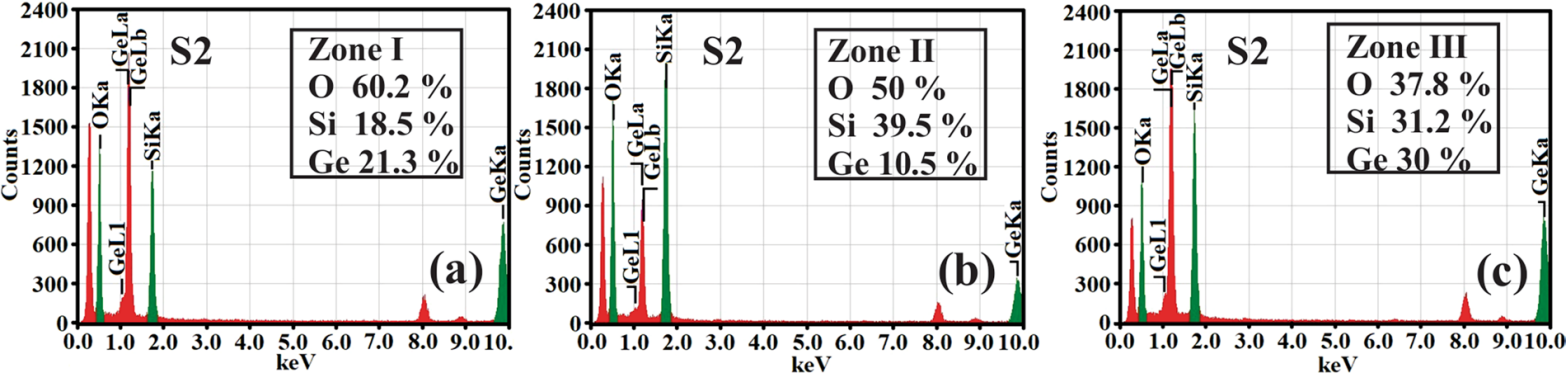
EDX spectra obtained from selected area in the three zones from Fig. 3a: (**a**) zone I at the top of the film indicates the presence of an amorphous SiGe oxide; (**b**) zone II (under zone I) is rich in SiO_2_ and depleted in Ge content; (**c**) zone III, the largest one positioned at the bottom of the film contains SiGe NCs embedded in oxide matrix.

Figure 3a shows the XTEM morphology of the high Ge content film (S2) annealed at 800 °C for 10 min. In this case, the morphology is completely different from that of S1 film. At the top of the film one can see an amorphous layer (zone I) that is quite rich in Ge (21.3%) as it results from the EDX spectrum in Fig. 4a, followed in depth by a layer (zone II) with low Ge content of 10.5% (Fig. 4b) expanded over about 50 nm, containing only few SiGe NCs. In the lowest part of the film (zone III) there is a uniform distribution (Ge content in film 30% - Fig. 4c) of well crystalized SiGe NCs with sizes between 15 to 50 nm. The NCs bigger than 30 nm are faceted and contain lattice defects like stacking faults and nanotwins (see Fig. 3b).

**Figure 5:**
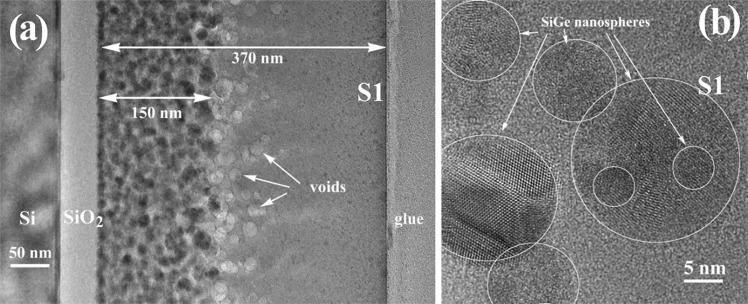
S1 film annealed at 1000 °C for 15 min: (**a**) low magnification XTEM image; voids are observed in the middle part of film; (**b**) high resolution detail of SiGe crystalline nanospheres.

Figure 5 shows a XTEM and HRTEM images taken on S1 film annealed at 1000 °C. In this case the SiGe NCs with sizes ranging between 5 and 25 nm are formed (Fig. 5b). They are spherical and look to be polycrystalline, showing several nucleation regions. The total film thickness is 370 nm. The amorphous top part of the film (free of SiGe NCs) is now larger to about 200 nm. One can observe a quite high density of nanovoids in the bottom part of the amorphous layer, at the interface with the region where the SiGe NCs appear. These nanovoids, with diameters between 10 and 20 nm, are formed by GeO gas accumulation. They are formed as this region is too far from the film surface and the GeO gas cannot be eliminated resulting in the formation of voids and consequently in film thickness expanding from 320 to 370 nm.

One can observe that all annealed S1 films have similar morphology except some differences due to the increase of the annealing temperature. In the case of high content Ge films (S2) the film morphology is however qualitatively different, the SiGe NCs nucleation and growth mechanism change due to the higher degree of Ge supersaturation in the oxide matrix.

**Figure 6:**
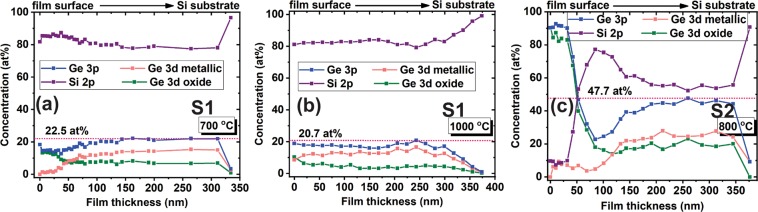
XPS depth profiles of S1 films with 700 °C RTA for 15 min (**a**), 1000 °C RTA for 15 min (**b**) and S2 films with 800 °C RTA for 10 min (**c**). The curves of atomic compositions in function of depth are given for total Ge, total Si, Ge_*metallic*_ and Ge_*oxide*_.

In Fig. 6 are presented the results obtained from XPS measurements, namely the atomic compositions of total Ge and Si, and also of metallic and oxidized Ge in the depth of S1 films annealed at 700 °C (a), at 1000 °C (b) and of S2 films annealed at 800 °C (c). For this, the films were etched in steps at different depths. One can see that in the 700 °C annealed S1 film, almost all Ge at the film surface is oxidized (down to ~50 nm) and then in depth the concentration of metallic Ge becomes higher than that of oxidized Ge and remains constant on the whole film thickness (Fig. 6a). In the sample S1 annealed at 1000 °C (Fig. 6b) the concentration curves have similar behavior with those in 700 °C RTA samples but Ge concentration is smaller, showing the Ge loss^5,38,40^. In the sample S2 with high Ge content (Fig. 6c), Ge is almost completely oxidized at the film surface, and then in depth, Ge is in metallic state but the concentration strongly decreases down to about 75 nm by Ge loss. For all samples, at depth bigger than 330 nm Ge signal disappears and the Si substrate is evidenced (Si2p peak at 99.7 eV).

**Figure 7:**
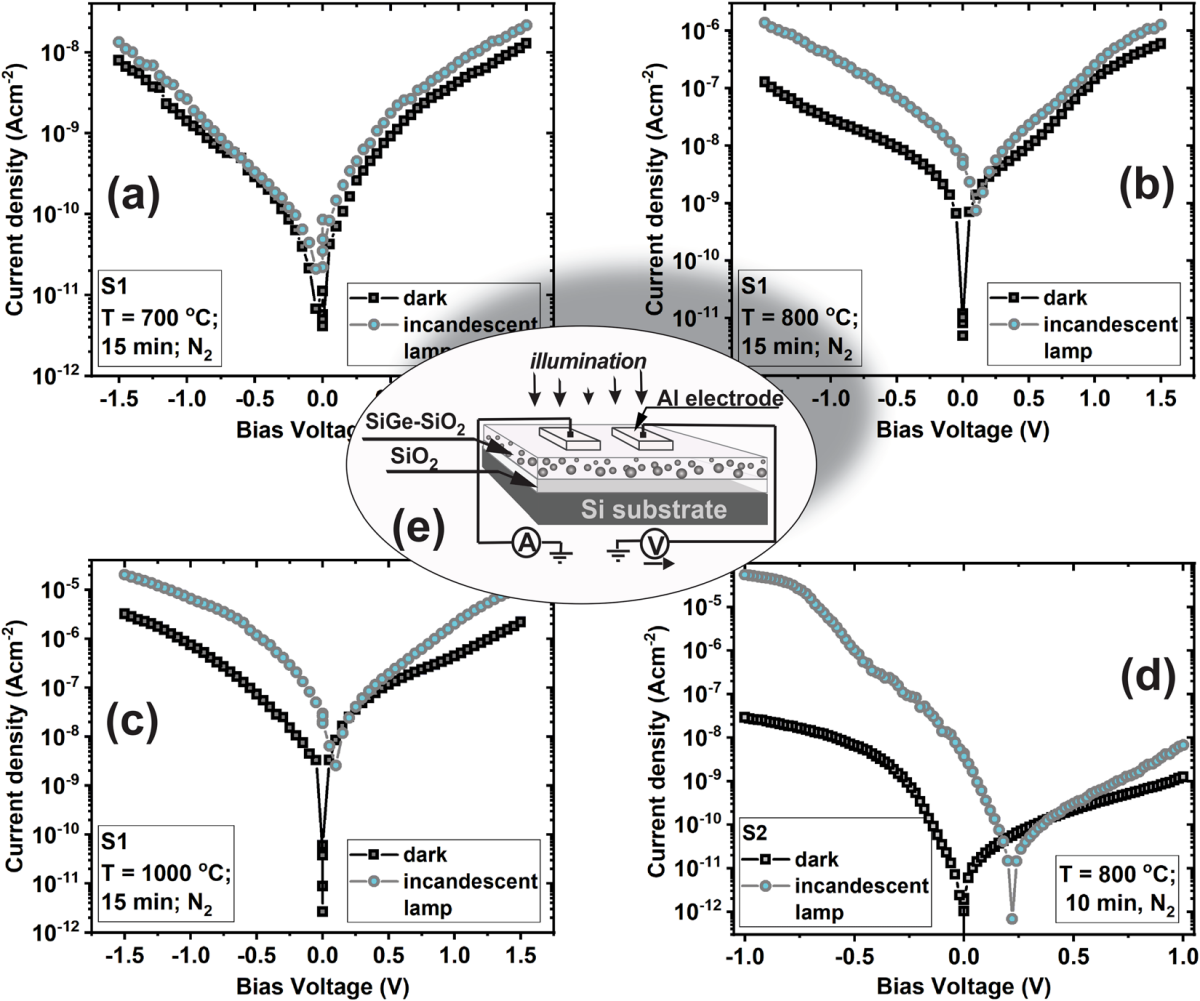
*J-V* characteristics obtained in dark and under illumination with an incandescent lamp on S1 (25%Si25%Ge:50%SiO_2_) film annealed at (**a**) 700 °C, (**b**) 800 °C and (**c**) 1000 °C and on S2 (5%Si45%Ge:50%SiO_2_) film annealed at (**d**) 800 °C. (**e**) Schematic representation of the investigated structures and measurement setup.

To see the influence of the annealing temperature on the photosensitivity of the films, current - voltage measurements in dark and under illumination with an incandescent lamp (20 W) were performed on samples with a planar configuration of electrodes. The incident optical power which falls on the active area of sample is 20 mWcm^−1^ measured by using a Thorlab power-meter PM100D coupled with a thermal power sensor S401C. Figure 7 displays the characteristics of current density versus voltage (*J - V*) recorded in dark and under illumination on S1 films annealed at temperatures of 700 °C (a), 800 °C (b), 1000 °C (c) and on S2 film annealed at 800 °C (d). Also, in Fig. 7e a schematic representation of the investigated samples together with measurement setup are presented. The samples were biased in the range of −1.5 V ÷ +1.5 V for S1 films and in the range −1 V ÷ +1 V for S2 films. The *J - V* characteristic taken in dark on sample S1 RTA annealed at 700 °C is almost symmetrical and with the increase of annealing temperature up to 1000 °C it becomes low rectifying (Fig. 7(a–c)). The *J-V* characteristic measured on sample S1 RTA 700 °C (Fig. 7a) shows a small difference between the dark current characteristic and that under illumination on both voltage polarities.

On the contrary, the *J-V* curves obtained on 800 and 1000 °C RTA S1 films show a significant increase of the photocurrent density in respect to the dark current density, i.e. with about one order of magnitude for 800 °C RTA sample (Fig. 7b) and a little smaller increase for 1000 °C RTA ones (Fig. 7c). In Fig. 7d are presented the *J-V* curves taken in dark and under illumination on sample S2 annealed at 800 °C RTA. These characteristics are very different than those measured on S1 sample, in the sense that the dark current density is lower than for S1 800 °C RTA, probably due to Ge loss. Also, these *J-V* characteristics show a very strong rectifying behavior due to the interface between the bottom layer in the zone III with big SiGe NCs and SiO_2_ buffer layer. We have to remark that the photosensitivity is very much increased, the photocurrent density is higher with more than three orders of magnitude than the dark current density on the reverse branch.

**Figure 8:**
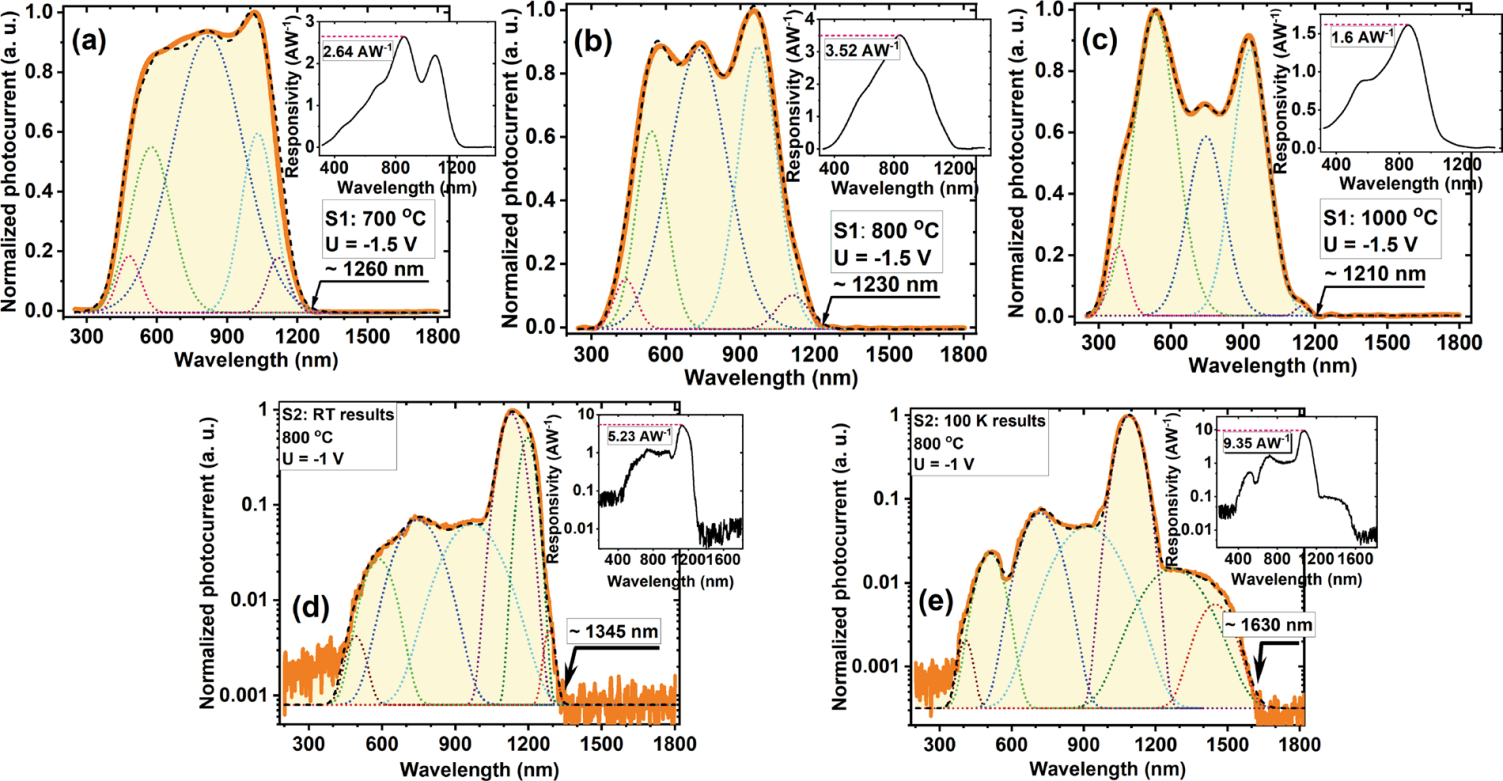
Normalized photocurrent spectra taken on S1 samples biased at −1.5 V (**a**) 700 °C, (**b**) 800 °C and (**c**) 1000 °C RTA for 15 min. Also, photocurrent spectra taken on S2 samples annealed at 800 °C for 10 min and biased at −1 V: (**d**) measured at room temperature (RT) and (**e**) at 100 K. The frequency of modulated light was 80 Hz. Each graphic contains as inset the corresponding spectral responsivity.

 Figure 8 presents the photocurrent spectra of the S1 samples annealed at 700 (a), 800 (b) and 1000 °C (c). The curves were normalized to the incident light intensity and to their maxima. Also, each graphic presented in Fig. 8a–e contains as inset the corresponding spectral responsivity. One can see that all spectra are broad. For samples S1 depending of the annealing temperature the spectra taken at RT spread between 300 nm and 1260 nm in SWIR while for samples S2 measured at RT between 400 nm and 1345 nm and expands to 1630 nm at 100 K. One can observe a relative change of the peak intensity that is dependent on the annealing temperature and also a shift of cutoff wavelength to higher energies with RTA temperature increase. This can be explained as follows: (i) the maximum positioned at 1100 nm (the peak resulting from deconvolution) is given by the contribution of Si substrate by surface photovoltage and gating effect^41^ (by capacitive coupling); (ii) the photocurrent peak at longer wavelengths than 1100 nm is due to the contribution of GeSi NCs (according to the film morphology); (iii) the photocurrent with the main maximum positioned between 710–750 nm (depending on RTA temperatures) is given by the contribution of defects present inside the film that are located at the interface of GeSi NCs / SiO_2_ matrix. In the case of sample S1, the shift of cutoff wavelength from ~1260 nm (in SWIR) to ~1239 nm when RTA temperature increases from 700 to 800 °C (samples S1) is due to both Ge oxidation^5^ according to XPS results and to Ge loss during annealing at higher temperatures. By further increasing the annealing temperature to 1000 °C, the cutoff wavelength shifts again to lower value of ~1210 nm by stronger Ge oxidation and loss that are supported also by XTEM and HRTEM images (Fig. 5).

We determined corresponding photoresponsivities from the recorded spectral photocurrents for all samples (insets in Fig. 8). So, for sample S1 annealed at 700 °C the maximum responsivity is 2.64 AW^−1^ that increases to 3.52 AW^−1^ for 800 °C RTA. By further increase of annealing temperature to 1000 °C, the photoresponsivity significantly decreases to 1.6 AW^−1^.

In Fig. 8d,e are presented the photocurrent spectra also normalized to the incident light intensity and to their maxima, measured on samples S2 800 °C RTA at RT (a) and 100 K (b). The spectrum taken at RT has a cutoff wavelength extended to 1345 nm in SWIR, while at 100 K it extends deeper in SWIR at 1630 nm. The cutoff extension for sample S2 in respect to sample S1 is due to the composition of SiGe NCs rich in Ge, in fact this contribution is given by SiGe NCs. For sample S2 800 °C RTA measured at room temperature and 100 K, we have obtained high photoresponsivities of 5.23 AW^−1^ and 9.35 AW^−1^. Regarding the mechanism, to the photocurrent contribute both photogenerated careers in the SiGe:SiO_2_ layer (SiGe NCs and/or defects) and those careers (holes) injected from Si substrate. The defects at the inteface SiGe NCs/SiO_2_ matrix can act as non-radiative recombination centers, producing the maxima in the range 710–750 nm, but also can trap photoholes and thus incresing the electron lifetime^5^.

## Conclusions

In this work we prepared two types of films of SiGe NCs embedded in SiO_2_ matrix by magnetron sputtering followed by RTA having high photosensitivity in VIS-SWIR. The SiGe:SiO_2_ composition during deposition is 50:50, while Si:Ge ratio is 25:25 vol% for samples S1 and 5:45 vol% for samples S2. RTA at 700, 800 and 1000 °C was performed. Samples S1 annealed at 700 and 800 °C have similar morphology, i.e. they consists of a bottom zone with SiGe NCs (5–8 nm size) and a top zone free of NCs. At 1000 °C RTA the film morphology is different, so the bottom zone is thinner and contains polycrystalline bigger SiGe NCs (5–25 nm). This film contains voids located between bottom and top zones that produce the film expansion. In the 700 °C RTA films S1, Ge concentration (metallic and oxidized) is relatively constant in the depth, excepting the film surface were it is smaller and almost all Ge is oxidized. In the rest of film the metallic Ge concentration is higher than the oxidized Ge one. In 1000 °C RTA sample S1, Ge concentration is a little bit smaller, showing the Ge loss. The morphology of 800 °C RTA film S2 shows three zones in the depth, i.e. a top amorphous zone of SiGe oxide rich in Ge, a middle zone depleted in Ge and with few SiGe NCs and a bottom zone with bigger SiGe NCs (15–50 nm), those bigger than 30 nm being faceted and contain stacking faults and nanotwins.

*J-V* characteristics in dark are almost symmetrical for 700 °C RTA film S1 and they become low rectifying with temperature increase (up to 1000 °C). *J-V* characteristics taken under illumination show similar behavior, but the photocurrent density is significantly higher than the dark current density, namely with approximately one order of magnitude for 800 °C RTA S1 and 1000 °C RTA S1. On the contrary, 800 °C RTA S2 presents strongly rectifying *J-V* characteristic in dark due to the interface between zone III and SiO_2_ buffer layer. The photocurrent density is remarkably higher with more than three orders of magnitude than the dark current density on the reverse branch. Photocurrent spectra taken on S1 samples with RTA at 700, 800 and 1000 °C show that S1 samples are photosensitive in VIS-SWIR (from 300 to more than 1250 nm). The cutoff wavelength is shifted from ~1260 to ~1210 nm when RTA temperature increases from 700 to 1000 °C, this being due to Ge oxidation and loss. The deconvolution spectra present maxima positioned at similar wavelengths and having different relative intensities for the S1 samples annealed at 700, 800 and 1000 °C. The maximum positioned at 1100 nm is due to the Si substrate contribution by surface photovoltage and gating effect, while the photocurrent at wavelengths longer than 1100 nm is due to the contribution of SiGe NCs. The photocurrent with the main maximum positioned at 710–750 nm results from the defects located at SiGe NCs / SiO_2_ interface. We obtained high photoresponsivities of 2.64 AW^−1^ for S1 annealed at 700 °C and 3.52 AW^−1^ for that annealed at 800 °C. For 1000 °C S1, the photoresponsivity significantly decreases to 1.6 AW^−1^. Photocurrent spectra taken on samples S2 800 °C RTA were measured at room temperature and at 100 K and their wavelength cutoffs are extended in SWIR to ~1345 nm and ~1630 nm, respectively, due to the contribution of Ge-rich SiGe NCs. The photoresponsivity is much higher, being 5.23 and 9.35 AW^−1^ at room temperature and 100 K, respectively. One can conclude that our films formed of SiGe NCs embedded in SiO_2_ have a great potential to be used in discrete optical sensors or integrated photodetectors including their integration with other materials in hybrid devices.

## Methods

### Preparation of SiGe NCs embedded in SiO_2_ thin films

The thin films with thickness of about 350 nm were deposited by magnetron co-sputtering from three independent targets of Si, Ge and SiO_2_ on cleaned n-Si substrate with 15–20 Ωcm resistivity. The volume concentration of each material (Si, Ge and SiO_2_) inside the film was obtained by adjusting the applied DC and RF power on target (P_*Si*_: 8–40 W, P_*Ge*_: 15–28 W and P${}_{Si{O}_{2}}$: 140–180 W). Prior to deposition, the main chamber was pumped down to 1 × 10^−7^ mTorr, and during deposition the work pressure were fixed at 4mTorr using Ar flux of 25 sccm. Films with two SiGe volume compositions in the SiO_2_ were deposited, one type with the same Ge concentration as Si one and the other with highier Ge concentration with the aim to control the Ge loss by fast diffusion and oxidation^42^. The films were denoted with S1 for SiGe:SiO_2_ ones with composition of 25%Si25%Ge:50%SiO_2_ and with S2 for SiGe:SiO_2_ films with 5%Si45%Ge:50%SiO_2_. A 30–50 nm SiO_2_ buffer layer was thermally grown on Si substrate for eliminating electrical contribution from the Si substrate and avoiding possible currents leakage. After deposition, the samples were annealed by RTA under flowing N_2_ gas at temperatures of 700, 800 and 1000 °C. Al coplanar contacts of 3 mm  ×  2 mm were deposited (after RTA of the films) by thermal evaporation and an active area of 6 mm^2^ between electrodes was obtained. The configuration of the samples is (SiGe-NCs:SiO_2_/SiO_2_/Si) and the photo- electrical measurements were performed using planar geometry.

### Structural, morphological and photo- electrical characterization

The morphology of films was investigated by cross section transmission electron microscopy (XTEM) using a JEOL analytical atomic microscope (JEM ARM 200F) and X-ray Photoelectron Spectroscopy (XPS) (SPECS equipment together with a PHOIBOS 150 analyzer). For electrical measurements, we used a setup consisting of electrometer (Keithley 6517A), temperature controller (LakeShore 331) and cryostat (Janis, CCS-450). The photocurrent - voltage characteristics were measured by using an incandescent tungsten halogen lamp of 20 W, and the photocurrent spectra were recorded by illuminating the samples with an incandescent quartz tungsten halogen lamp of 100 W coupled with a mechanical chopper (SR540), monochromator (MS257), suitable filters and a lock-in amplifier (SR830). The incident optical power on the investigated samples were measured using a Thorlab power-meter. The beam was collimated and the optical power that falls on the active area between electrodes was established by considering the two-dimensional overlap between the measured optical beam profile and the measured area for each sample.
